# Differential composition of the pulmonary microbiome in HIV-positive versus HIV-negative patients with *Pneumocystis jirovecii*

**DOI:** 10.1371/journal.pone.0334220

**Published:** 2025-10-10

**Authors:** Chang-Run Zhang, Mei Wang, Lei Wang, Li Liu, Yi-Dan Shen, Rui-Fang Chen, Shuai Ren, Wei-Zhong Jin, Hai-Cheng Tang, Qing-Guo Wu

**Affiliations:** 1 Department of Respiratory and Critical Care Medicine, Public Health Clinical Center, Fudan University, Jinshan, Shanghai, China; 2 Department of Infection and Immunology, Shanghai Public Health Clinical Center, Fudan University, Jinshan, Shanghai, China; Dognosis India Private Limited, INDIA

## Abstract

*Pneumocystis jirovecii* is frequently detected in HIV patients and individuals with compromised immune function. The clinical outcomes of these two groups differ significantly, yet the underlying reasons remain unclear, with limited studies addressing this issue. This study investigates the alterations in the pulmonary microbiota of HIV-positive and non-HIV patients following *pneumocystis jirovecii* infection.Collect bronchoalveolar lavage fluid from patients with HIV and non HIV infected *Pneumocystis jirovecii*, and compare the differences in pulmonary microbiota between the two groups.In total, 77 patients with pulmonary infection that had next generation sequencing performed on their bronchoalveolar lavage fluid and confirmed *pneumocystis jirovecii* infection were recruited in our study. Of the 77 patients with *pneumocystis jirovecii* infection, 52 were infected with HIV, and 25 were uninfected.Our findings indicate that HIV-positive patients exhibit a more diverse microbiota, predominantly characterized by viral co-infections. Specifically, 88.5% of HIV-positive patients experienced viral co-infections, primarily involving herpes viruses, followed by bacterial (61.5%) and fungal (40.4%) co-infections. In contrast, non-HIV patients predominantly exhibited bacterial co-infections (72%), followed by viral (52%) and fungal (36%) co-infections. By analyzing the next generation sequencing data of both groups, we identified statistically significant differences in viral infections (p < 0.001), while no significant differences were observed for bacterial or fungal infections. Furthermore, among the background bacteria detected via next generation sequencing in both patient groups, 22 microbial species were commonly present. Notably, *Leptospiral virus*, *Rosette fungus*, and *Actinomycetes* were detected at higher frequencies in HIV-infected *pneumocystis jirovecii* patients, with statistically significant differences.Through comparing the pulmonary microbiota profiles of HIV-positive and non-HIV patients post-*pneumocystis jirovecii* infection, we uncovered distinct differences between the two groups, which may hold implications for guiding subsequent treatment strategies and improving clinical outcomes.

## Introduce

*Pneumocystis jirovecii* (PJ) is a well-known opportunistic infection that has been found worldwide and is primarily transmitted from person to person through the inhalation of particles in the air [1]. *PJ* can lead to pneumocystis jirovecii pneumonia (PJP), which is common in patients with human immunodeficiency virus (HIV), but the incidence of PJP in this population has significantly decreased in recent years due to early HIV treatment [2]. However, the incidence of PJP in non-HIV patients has increased in recent years, which is due to the use of immunosuppressive drugs, anti-cancer drugs, and the increase in the number of organ transplant recipients [3]. *PJ* infections in HIV patients usually progress to a subacute course, while *PJ* infections in non-HIV patients usually progress rapidly [4]. Although Trimethoprim-Sulfamethoxazole (TMP-SMX) is still recommended as first-line treatment for PJP regardless of HIV status [5], the mortality rate of PJP in non-HIV patients (30–60%) is significantly higher than that of PJP in HIV patients (10–20%) [6]. The difference in mortality rates indicates that non-HIV PJP may have different pathogenesis and immune abnormalities from HIV PJP.

For a long time, people have believed that the lungs are a sterile environment. However, with the development of molecular research, people have discovered that there are many microbial sequences in the lungs [7]. Given the diversity of microbial communities in the lungs, coupled with the similar origins and structures of the gut and lungs, it is generally believed that there is a certain correlation between the microbial community in the lungs and the host immune response [8,9], and research in asthma [10], lower respiratory infections [11], and tuberculosis [12] has been conducted accordingly. In PJP patients, researchers found no significant difference in the microbial detection in the alveolar lavage fluid between PJP patients and non-PJP patients [13], but some studies have found that the microbial diversity in PJP patients’ lungs decreased compared to non-PJP patients, and the Firmicutes play a crucial role in the negative regulation of the MAPK signaling pathway in PJP [14]. The earliest study comparing the lung microbiome of HIV-infected individuals and uninfected individuals found that the abundance of the Proteobacteria phylum increased in the uninfected group, while the Actinobacteria, Bacteroidetes, and Firmicutes phyla all increased in the HIV-infected group, with Prevotella being significantly increased [15]. Although there have been more studies comparing the lung microbiome differences between PJP and non-PJP, HIV and non-HIV, there is currently no research on the microbial differences between the co-infection conditions.

In summary, there is a lack of research on the pulmonary microbiome in patients with and without HIV infection when infected with *PJ*. This study aims to clarify the difference in pulmonary microbiome between these two groups of patients by analyzing the results of next-generation sequencing of bronchoalveolar lavage fluid from both groups, thereby laying a foundation for subsequent studies on pathogenesis and other related issues.

## Methods

### Subjects

We collected patients infected with *PJ* in the Department of Respiratory and Critical Care Medicine and the Department of Infectious Immunology of Shanghai Public Health Clinical Center from 1,Jan,2022–31,Dec, 2024. Inclusion criteria: detection of *PJ* in bronchoalveolar lavage fluid(BALF) next-generation sequencing(NGS) results. This study was performed in line with the principles of the Declaration of Helsinki. Approval was granted by the Ethics Committee of Shanghai Public Health Clinical Center (Dtae:2025-7-23/No:2025-S092-01). And due to reasons such as anonymous data analysis, the Ethics Committee agreed to waive the requirement for informed consent.

### Data collection

Clinical data were retrospectively acquired from medical records(1,Jan,2022–31,Dec 2024), encompassing patient age, sex, cancer history, steroid usage history, CD4 + T lymphocyte counts, clinical outcomes, and NGS findings.

### BALF sample processing and DNA extraction

For each participant enrolled,1.5 to 3.5 mL of BALF was collected according to standard procedures.The collected BALF were liquefied 10 min at 65°C. Glass beads and lysozyme were added to the sample. After mixing and shaking, and DNA was extracted using the TIANamp Micro DNA Kit (DP316, Tiangen Biotech) according to the instructions.

### Library construction and sequencing

The extracted DNA was subjected to fragmentation, terminal repair, connector connection and PCR amplification for library construction. Agilent 2100 Bioanalyzer was applied for quality control to make sure that the size of fragments in the constructed DNA library reached up to 300 bp., and the concentration of the library using Qubit dsDNA HS Assay Kit (Thermo Fisher Scientific, USA). Then, the quality confirmed library is sequenced through the BGISEQ-200 platform.

### Sequencing data analysis

To control the impact of contamination, negative controls and positive controls were prepared in parallel and sequenced in the same operation. Low-quality, low-complexity and short reads (length<35 bp) were removed by using in-house software. high quality data was aligned to the human genome to remove human reads, and the remaining sequencing data was simultaneously aligning to the NCBI-based microbial reference database established by knoindx TM, so as to achieve a taxonomic classification to each sequence read for microbial identification. The in-house built reference database contained 10216 bacteria, 5875 viruses, 3789 fungi and 432 parasites.

### Criteria for positive NGS results

The criteria for positive NGS results were defined as followsm, (i)Mycobacterium tuberculosis and key fungi, any sequence number was detected on the premise of excluding contamination.(ii)The sequence number of key bacteria ≧5 was considered as responsible pathogenic bacteria, otherwise, the background was considered.(iii)Virus sequence number ≧2 was considered as responsible pathogen, otherwise was considered as radiation background.(iv)Skin colonizers or environmental colonizers are generally not considered as responsible pathogens if they are not detected in large numbers.

### Statistical analysis

Statistical analyses and graphing were performed using SPSS 21.0 and Prism 10. Continuous variables were denoted as medians, and categorical variables were denoted as proportion (%). Continuous variables that conformed to the normal distribution were analyzed by group t test. The comparisons of lung microorganisms between two groups were performed by Pearson chi-square test or Fisher’s exact test. A *P* value of <0.05 was considered significant.

## Result

### Patient characteristics

In total, 77 patients with pulmonary infection that had NGS performed on their BALF and confirmed *PJ* infection were recruited in our study.Of the 77 patients with *PJ* infection, 52 were infected with HIV, and 25 were uninfected.Regardless of HIV infection or not, the proportion of male patients was higher than that of female patients, and the difference was statistically significant (p = 0.03). In terms of age, the average age of non-HIV patients was significantly higher than that of HIV patients, and the difference was statistically significant (p < 0.001). In terms of CD4^+^T cells, as we know, HIV patients had a very low CD4^+^T cell count (average 34cell/ul), while non-HIV patients also had CD4 + T cell reduction, but it was still significantly higher than HIV patients (p < 0.001). In terms of influencing the body’s immune status, non-HIV had a higher proportion of patients, among whom 24% had a history of tumors and 20% had a history of hormone use, which were significantly higher than those of HIV group, and the differences were statistically significant (p < 0.001). Characteristics of HIV-infected and uninfected patients with *PJ* infection are shown in Table 1. (The original sequencing results and clinical indicators are provided in S1 File).

**Table 1:** Comparison of the characteristics of PJ infection between non-HIV and HIV patients.

Patient characteristics	HIV-infected	HIV-uninfected	P value
	patients (n = 52)	patients (n = 25)	
Age (yrs, median)	46.3	61.5	<0.001
Male, n (%)	48 (92.31%)	18 (72%)	0.03
CD4 + T cell count (cell/uL, median)	34	291	<0.001
History of Cancer, n (%)	2 (3.8%)	6 (24%)	<0.001
Hormonal history, n (%)	0	5 (20%)	<0.001
Death, n (%)	3 (5.7%)	1 (4%)	0.74

### Lung microbiomes detected by NGS in non-HIV with *PJ*

**Fig 1:**
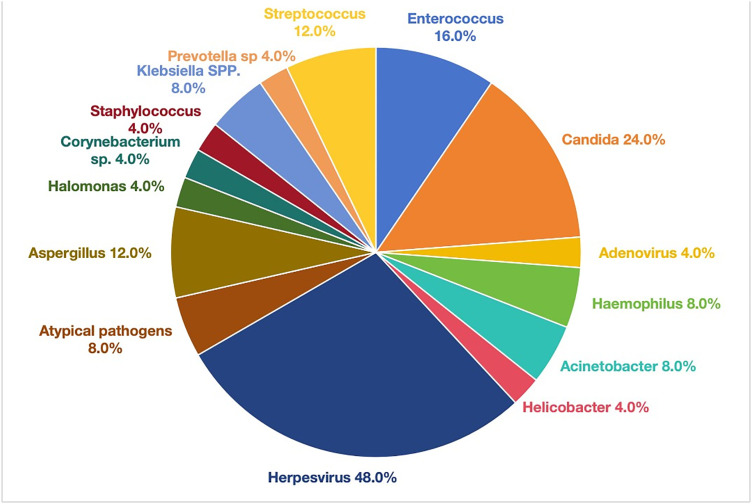
The positive results of microorganisms in BALF of non-HIV patients after *PJ* infection.

**Fig 2:**
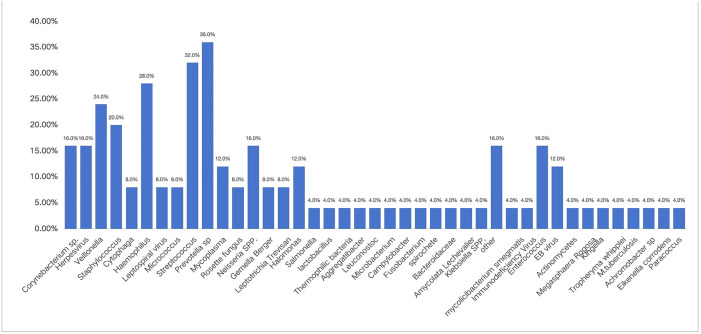
The background of microorganisms in BALF of non-HIV patients after *PJ* infection.

A total of 42 kinds of microorganisms were detected in the BALF of non-HIV with *PJ* patients, among which bacteria accounted for 72%, viruses accounted for 52%, fungi accounted for 36%, and atypical pathogens accounted for 8%. Among all kinds of pathogens, Enterococcus (16%), herpes virus (48%), and Candida (24%) ranked the first in the infection rate (Fig 1). In non-HIV with *PJ* patients, 98 kinds of microorganisms were detected in the background microorganisms, mainly bacteria, followed by viruses, among which Prevotella accounted for the highest proportion of bacteria (36%), and the detected viruses included herpes virus, lepto virus, EB virus, and immunodeficiency virus, with low proportions (Fig 2).

### Lung microbiomes detected by NGS in HIV with *PJ*

**Fig 3:**
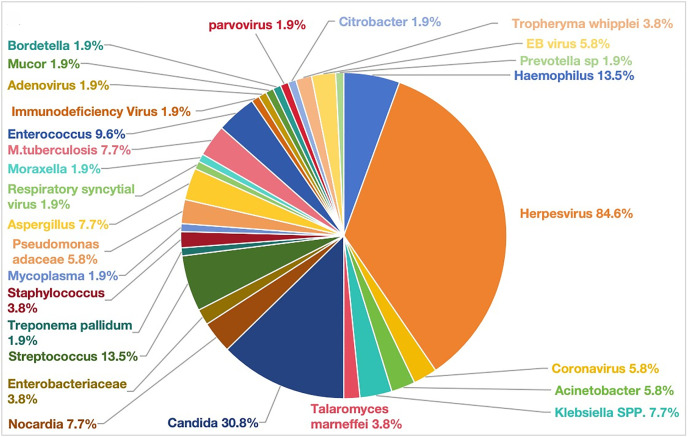
The positive results of microorganisms in BALF of HIV patients after *PJ* infection.

**Fig 4:**
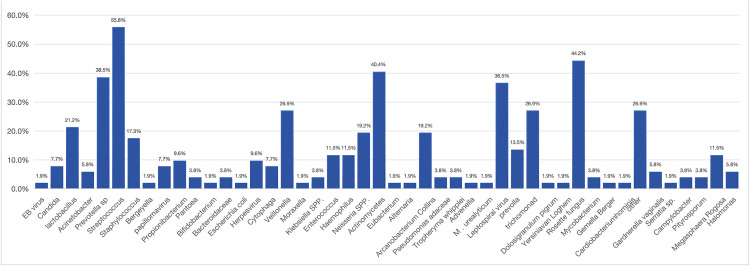
The background of microorganisms in BALF of HIV patients after *PJ* infection.

In patients with HIV and *PJ*, a total of 126 pathogens were detected in BALF, with viruses predominating, with approximately 88.5% of patients co-infected with viruses, mainly herpesviruses (84.6%), the incidence of bacterial infection was significantly lower in HIV patients than in non-HIV patients, at only 61.5%, with streptococcus and hemophilus being the main pathogens (13.5%); moreover, HIV patients were more likely to co-infect with fungi (40.4%), with common candida being the main pathogen (30.8%) (Fig 3). In terms of background bacterial species, the diversity of background microorganisms in HIV patients was more complex, with a total of 280 microorganisms detected, with rossella (44.2%), actinomyces (40.4%), streptococcus (55.8%), and prevotella (38.5%) being the most common (Fig 4).

### Differences in lung microbes between non-HIV and HIV patients with *PJ* infection

**Fig 5:**
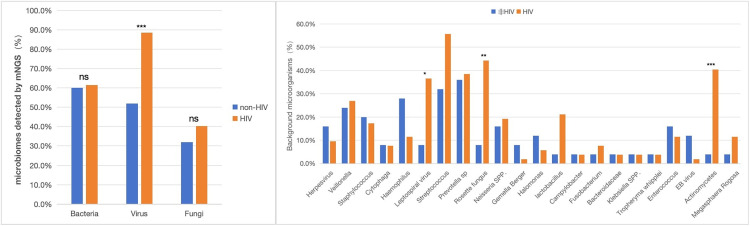
(Left) Comparison of lung microbial species detected by NGS between HIV infected and non-HIV infected patients with *PJ*; (Right) Comparison of the same background; ns, no sense, *, P < 0.05; **, P < 0.01,***,p < 0.001).

By performing NGS on the lavage fluid, we found that a greater number of microbial species were detected in HIV-Infected *PJ* patients compared to non-HIV-Infected *PJ* patients.On the premise of infecting *PJ*, compared with non-HIV patients, HIV patients were more likely to be infected with viruses (52% vs 88.5%), and the difference between the two groups was statistically significant (p < 0.001). In terms of bacteria and fungi, although the infection rate of HIV patients was higher than that of non-HIV patients, there was no statistically significant difference (Fig 5 left).In addition to the positive results detected by NGS, We also found that there are some differences in the background microorganisms in the lungs of non-HIV and HIV patients after *PJ* infection. By detailed analysis of the bacteria in the background microbes, we found that 22 microorganisms were present in both types of patients. Among them, the HIV infected *PJ* patients had a higher detection rate of Leptospiral virus, Rosette fungus, and Actinomycetes (Fig 5 right).

## Discussion

PJP began to rise during the HIV epidemic in the 1980s. With the effective control of HIV, the incidence of PJP gradually decreased, but with the increase of organ transplantation and immunodeficiency diseases, the number of non-HIV infected PJP patients gradually increased [16,17], and related studies have found that the prognosis of non-HIV infected PJP patients is worse than that of HIV patients [6]. However, in this retrospective study, it was found that the mortality of HIV patients infected with *PJ* was slightly higher than that of non-HIV patients, but the difference was not statistically significant, which may be related to the large sample size gap in this study.Furthermore, this study found that *PJ* infection was more common among male patients, regardless of whether they were HIV-infected or not, suggesting a gender bias. In terms of patient baseline conditions, non-HIV patients with *PJ* often had tumors or long-term use of hormones, which were immunosuppressive conditions consistent with current risk factors for PJP, and as the number of immunosuppressed individuals in society increases, the incidence of *PJ* infection is likely to rise annually. Therefore, it is crucial to clarify the progression mechanism and immune dysregulation of *PJ* or PJP.

The increasing incidence of PJP without HIV infection has prompted us to pay attention to PJP research again. At present, with more and more attention paid to the importance of microbes in the occurrence and evolution of diseases, many researchers have begun to study the changes of lung microbiome in the occurrence of lung diseases in order to find better treatment options, including comparative studies of PJP infection or not [18] and HIV infection or not [19]. As an opportunistic pathogen, *PJ* is commonly found in HIV or partially immunocompromised patients. Patients infected with *PJ* are often complicated with other microbial infections, which is closely related to the patient’s immune status. By analyzing the changes of lung microorganisms in patients infected with *PJ*, it is helpful for us to better identify the disease and guide clinical treatment. This study is the first to compare the difference of lung microbiota between non-HIV and HIV patients infected with *PJ*, and found that there are significant differences in the background microbial composition between the two groups, which provides important reference for future research on pathogenesis and clinical treatment. For example, in the background microbiota of HIV patients, there are more fungi and viruses. In the context of HIV-induced immune deficiency, the background microbiota may become pathogenic at any time due to changes in immune status. In clinical treatment, preventive antifungal or antiviral therapy may have better clinical outcomes. In non-HIV patients, the background microbiota is dominated by bacteria, so the early use of broad-spectrum antibiotics may inhibit disease progression. These views need further clinical verification.

Our study was based on the premise of *PJ* infection, that is, the patient was basically determined to be in the immunosuppression state, and the microbial differences between non-HIV and HIV susceptible populations were compared. We found that compared with non-HIV patients, HIV patients are more likely to be infected with other microorganisms after *PJ* infection, and the background microbes are also more abundant, which may be related to the very low CD4^+^T cells in HIV patients. However, HIV patients usually have a better prognosis, which may be related to the restoration of CD4^+^T cell immune function after effective antiviral treatment [20]. However, in non-HIV patients infected with *PJ*, CD4^+^T cells are only slightly reduced, and the richness and positive rate of microbes in the lung are lower than those in HIV patients. However, clinical findings show that non-HIV patients infected with *PJ* often cause PJP and have rapid disease progression and poor prognosis [21], suggesting that non-HIV patients infected with *PJ* may have other immune disorders. For example, researchers have found that the polarization of alveolar macrophages in immunodeficient mice infected with PJP is changed. After immunodeficient mice infected with PJP, the alveolar macrophages are dominated by M1 phenotype. Immunosuppressed mice have increased clearance of pneumocystis and reduced inflammation [22], and similar results have been found in the bronchoalveolar lavage fluid of non-HIV PJP patients [14]. Therefore, studies on the immune system of non-HIV patients infected with *PJ* may clarify the cause and mechanism of rapid disease progression in these patients.

## Conclusion

This study analyzing NGS data from BALF demonstrated distinct differences in the lung microbial composition between HIV-negative and HIV-positive patients with *PJ*. HIV-positive patients exhibited greater diversity of commensal lung microbiota and a higher prevalence of co-infecting pathogens, predominantly viruses (with herpesviruses being most frequent). In contrast, bacterial co-infections were more common in HIV-negative patients. Notably, despite this increased microbial burden and diversity, HIV-negative *PJ* patients experienced worse clinical outcomes. This counterintuitive finding strongly suggests that factors beyond microbial load, such as underlying dysregulation of host immune responses in non-HIV immunosuppression, critically influence disease severity and prognosis, warranting further investigation.

## Supporting information

S1 File Supplementary excel. (XLSX)
